# Dublin

**Published:** 1912-11

**Authors:** 


### CORRESPONDENCE.

### Dublin.

The outlook for the progress of scientific medicine in Ireland is not promising. Many causes contribute to stay, or at least hinder original work in this country; not the least of these is politics and very intimately associated with this is religion.

There are two Irelands, a Protestant and a Roman Catholic; all the former are Unionists as are a considerable percentage of Roman Catholics, but the great majority of them are Home Rulers. These latter practically control all public appointments in three-fourths of the country, and of course they appoint to office candidates that hold the same political and religious views as they do. Indeed this same spirit controls all elections of medical officers throughout the country. This applies to the appointment to asylums, coroners, gaol medical officers, medical health officers and a number of minor appointments.

There is little incentive to study after qualification; and inducement to court popular favor by joining friendly and benefit societies such as the Masonic, the Corinthian, the Ancient Order of Hibernians, etc.

Then since the great majority of general medical practitioners shut their open shops and adopted society practice, we have a considerable number of physicians taking club practice at the rate of $1 a member, for attendance on the member, his wife, and family. Of this one dollar fee he gives half to a pharmaceutical chemist for medicine for each subscribing member. So many medical men in our midst pass their lives in this worse than Egyptian bondage.

By our unequalled Poor Law system of medical relief our poor are well provided for. The whole country is divided into dispensary districts and to each district a medical officer is appointed by the local authorities, subject to the approval by a central board in Dublin. The positions, as a rule, are keenly contested; the emoluments average £300 a year, and in some cases £400. The medical officer holds the appointment for life, subject to good behavior, and on retiring from office gets a pension equal to two-thirds of his income from the office. He is free to attend private patients, when same does not interefere with his dispensary duties; he also gets a month's holiday every year, during which time a substitute is paid to act for him. Hon. Mr. Lloyd George's National Insurance Bill is yet to be seen. It is very far reaching in its effects and so far as the act can be deciphered by a non-legal mind seems well calculated to bring trouble and entail expense on every householder.

Happily for the medical profession in Ireland the medical clauses of the act do not extend to this country. But our brethren in England and Scotland will be hard hit by the act. It is common knowledge that our medical brethren engaged in club practice in England have cruel taskmasters in the members of the club committees; and it was hoped that the incubus would be shaken off by the profession and its members freed from serfdom. But our present ministry is kept in power by a hetrogeneous conglomeration of log rollers, and the two strongest parties in the group are the Laborers and the Home Rulers. With one voice the Labor members demanded control of the medical offices and their friends the Home Rulers joined in the demand. Our great organization, the British Medical Association took active steps to protect the profession; its Journal, with its immense circulation and great influence, week after week expressed the contemplated injustice. A defense committee was formed and cooperated with the Council of the British Medical Association.

Hope revived in the breasts of the club doctors; subscriptions came in freely to the defense fund; subscribers came in for the Journal that was doing such good work; when to the astonishment of all, the Secretary of the Council of the British Medical Association, accepted, with the concurrence of the Council, an appointment under the Act. Arnold could claim that five brothers in arms were promoted over his head and that his feats at Saratoga were not duly honored. But our secretary was much honored and greatly trusted; and we had no more idea of his fastening the manacles on his brothers than Washington had that Andre was getting the plans of West Point.

Owing to the great pressure of business the government pushed the Act through the Commons without discussion; with the result that few can ever guess at the meaning of many of its clauses, and today many lecturers are travelling through the Kingdom trying to make plain the unintelligible Act, which came into force July 1, 1912.

A further criticism of this Act and the notice of the Blue Book on Vivisection I must unavoidably postpone until next month.

CLEOPAS.

